# Social aspects of collision avoidance: a detailed analysis of two-person groups and individual pedestrians

**DOI:** 10.1038/s41598-023-32883-z

**Published:** 2023-04-08

**Authors:** Adrien Gregorj, Zeynep Yücel, Francesco Zanlungo, Claudio Feliciani, Takayuki Kanda

**Affiliations:** 1 Okayama University, Okayama, Japan; 2 Osaka International Professional University, Osaka, Japan; 3 ATR International, Kyoto, Japan; 4 The University of Tokyo, Tokyo, Japan; 5 Kyoto University, Kyoto, Japan

**Keywords:** Engineering, Mathematics and computing

## Abstract

Pedestrian groups are commonly found in crowds but research on their social aspects is comparatively lacking. To fill that void in literature, we study the dynamics of collision avoidance between pedestrian groups (in particular dyads) and individual pedestrians in an ecological environment, focusing in particular on (i) how such avoidance depends on the group’s social relation (e.g. colleagues, couples, friends or families) and (ii) its intensity of social interaction (indicated by conversation, gaze exchange, gestures etc). By analyzing relative collision avoidance in the “center of mass” frame, we were able to quantify how much groups and individuals avoid each other with respect to the aforementioned properties of the group. A mathematical representation using a potential energy function is proposed to model avoidance and it is shown to provide a fair approximation to the empirical observations. We also studied the probability that the individuals disrupt the group by “passing through it” (termed as intrusion). We analyzed the dependence of the parameters of the avoidance model and of the probability of intrusion on groups’ social relation and intensity of interaction. We confirmed that the stronger social bonding or interaction intensity is, the more prominent collision avoidance turns out. We also confirmed that the probability of intrusion is a decreasing function of interaction intensity and strength of social bonding. Our results suggest that such variability should be accounted for in models and crowd management in general. Namely, public spaces with strongly bonded groups (e.g. a family-oriented amusement park) may require a different approach compared to public spaces with loosely bonded groups (e.g. a business-oriented trade fair).

## Introduction

Groups represent an important component of pedestrian crowds^1,2^, and lately they have been the subject of many studies, focusing on such themes as their effect on crowd dynamics^3^, the observation of their shape and velocity^1,4–9^, mathematical and computational modeling of interaction dynamics^5,10–15^, and dependence on social structure and interaction level^16–19^. Many works are based on ecological observations^1,4–6,12,16–21^, which may be argued to be indispensable in dealing with social aspects of human behavior^4,22–27^.

To plan safe buildings^28–32^ and manage crowds in busy public places (i.e. transportation hubs) and events^33^, it is important to consider group behavior in crowd simulators and monitoring/predicting tools^34–37^. This includes taking into account factors such as the structure of different types of groups, their social relations^38^, and cultural differences^39,40^ for more accurate simulations. It is also crucial to model not only the internal dynamics of the group, but also their reaction to the external environment, such as the presence of other pedestrians, while keeping in mind the aforementioned characteristics of each group. Furthermore, it is essential to consider the impact of the group on other pedestrians and how their behavior may be influenced by the presence of the group.

However, the majority of the studies mentioned above concentrate solely on the collective behavior of groups, i.e. the dynamics that drive the group to move as one cohesive unit. Mathematically oriented works on group dynamics may introduce the concept of a “group potential” and examine it by assuming that interactions with pedestrians outside the group can be approximated as white noise^5^ or as an external “mean-field” potential^12^ on average. Similarly, observation-based works^6,16,18,19^ often describe group properties using probability density functions defined by an average process that neglects the specifics of the environment.

On the other hand, when introducing group behavior into a microscopic simulator, it is essential to incorporate specific rules that describe interactions between the group and the environment, particularly with surrounding pedestrians. Introducing in-group dynamical rules is a logical starting point, and these can simply be added to the collision-avoidance rules used for individuals^41,42^. For instance, if a two-person group encounters a single-walking pedestrian in an acceleration-based or “Social Force” model^43–45^, the acceleration terms of the group’s pedestrians can be obtained by summing the individual-individual collision avoidance terms and those resulting from in-group interaction^46^. The behavior of the lone pedestrian can be modeled by adding the two avoidance terms with respect to the pedestrians in the group. In other words, collision avoidance is treated as a one-to-one behavior, group dynamics are regarded as an exclusively in-group phenomenon, and the overall dynamics is the sum of these distinct components.

This is the (classical) linear superposition principle^47^, which is typically assumed (and verified) in mechanics and simplifies physical models and their dynamics to a great extent. However, this principle does not necessarily apply to pedestrian dynamics. For instance, when a single walking pedestrian encounters a two-person group walking side by side, particularly if they are socially interacting, he or she may choose to avoid walking through them, even if it seems like the best choice from a pure collision-avoidance perspective. This intrusion decision would be likely, should the two pedestrians be perceived as independent.

In this work, we investigate a relatively unexplored aspect of pedestrian behavior and crowd dynamics: collision avoidance of (or against) groups, and in particular, its dependence on the groups’ social attributes, which refer specifically to social relation and intensity of interaction.

In doing that, we use two data sets of pedestrian trajectory including annotations of groups’ social attributes to investigate the nature of individual-group collision avoidance. Moreover, we focus particularly on groups composed of 2 people (i.e. dyads), since they are much more common than larger ones and they actually constitute their fundamental building block together with triads (i.e. for an easy navigation and social interaction, large groups break into sub-groups of 2 or 3 people)^4^. In addition, larger groups (of 3 or more people) may require a categorization of pair-wise social relations or interactions, which may be very complex to formulate or generalize. In that respect, we use the word *group* to refer simply to dyads. As groups’ counterpart in collision avoidance, we focus on *individuals*, which is a term we use to refer to people not appertaining to a group.

**Figure 1:**
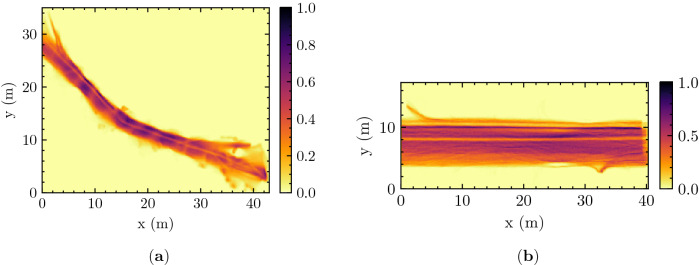
The normalized cumulative density maps for (**a**) the ATC data set and (**b**) DIAMOR data set. The environment is discretized as a 2D mesh with a grid cell size of 10 cm by 10 cm, and the number of observations is counted in each grid cell. Normalization refers to the scaling of this histogram with its maximum value.

**Table 1:** Number of groups annotated with each (a) social relation (in ATC data set) and (b) intensity of interaction (in DIAMOR data set).

(a)	
Social relation	# of annotations
Couples	69
Colleagues	314
Family	180
Friends	253
Total	816

(b)	
Intensity of interaction	# of annotations
0 (no interaction)	140
1	159
2	460
3 (strong interaction)	100
Total	859

## Methods

### Data sets

In this study, we used two data sets, namely the ATC data set and DIAMOR data set, both of which are reviewed and approved for studies involving human participants by the ATR ethics board^5,6^, are publicly available and contain trajectories derived from range data^48–50^. From these trajectories, we computed the normalized cumulative density maps of the experiment environments shown in Fig. 1.

The data sets are annotated based on video footage for different social attributes of groups. Specifically, the ATC data set is annotated from the viewpoint of social relations, whereas the DIAMOR data set is annotated from the viewpoint of the intensity of interaction.

For the ATC data set, possible options for social relation are couples, colleagues, family and friends, which are determined through the domain-based approach of Bugental^51^ and correspond to the domains of mating, coalitional, attachment and reciprocal, respectively. This annotation process yields the values presented in Table 1a.

For the DIAMOR data set, the intensity of interaction is evaluated at 4 degrees, 0 representing no-interaction and 1, 2, and 3 representing weak, mild and strong interaction, respectively. This annotation process yields the values presented in Table 1b. Note that in order not to bias the coders’ assessment, we only defined the number of interaction levels as 4, but we did not give any guidelines on what can be considered as weak, mild or strong interaction^52^. Instead, we let the coders grasp a feeling about different intensities of interaction through a free-viewing task (i.e. letting them watch the videos for 3 h before giving any labels, see Supplementary Information Section 1 for further details.).

### Approach

Provided that the group and the individual do not perform any collision avoidance, we can expect their (relative) motion to be approximated by a straight line. We are aware that this assumption requires implicitly the environment to be sufficiently straight and wide (e.g. like DIAMOR, see Fig. 1b and the discussion in Supplementary Information Section 7) and is valid up to a reasonable range (i.e. over a few meters). Namely, in environments with complex geometries (curved or with many obstacles, intersections etc.), the pedestrians need to deviate as part of their interaction with the boundaries. Similarly, over long distances, they will eventually meet some walls, or divert towards different goals, making their relative motion bent. Nevertheless, in a sufficiently straight corridor and on a scale of few meters, we can expect it to be a good approximation.

This trivial assumption can be considered to serve as a hypothesis, opposite to what we actually anticipate. Based on such a hypothesis, the deviation of (relative) motion from a straight line can be attributed to group-individual collision avoidance. Specifically, by measuring this deviation with respect to different social relations or intensities of interaction (of the group), we may understand the reflections of such group attributes on collision avoidance.

**Figure 2:**
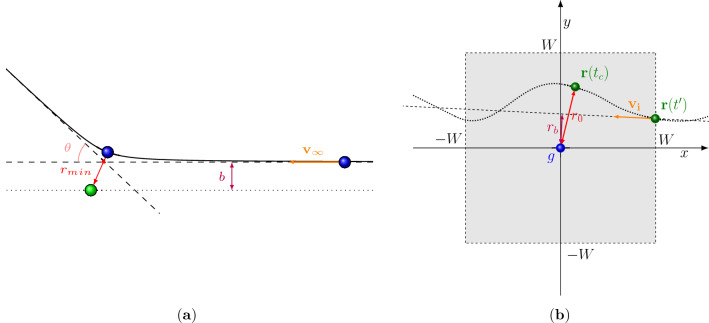
(**a**) Illustration of the scattering problem in physics. A mobile particle (in blue) is projected toward a fixed particle (in green). The impact parameter, *b*, is the straight-line distance between the particles, and $r_{min}$ is the closest approach. The particle is deflected with an angle $\theta$. (**b**) A typical pedestrian avoidance situation in the *group-centered reference frame*. The individual enters the vicinity of the group (gray region) at time $t^{\prime }$ with velocity ${\textbf{v}}_i$. The straight-line distance from the individual to the group is denoted by $r_b$. At time $t_c$, the individual is closest to the group at a distance of $r_0$.

This formulation presents a striking resemblance to one of the fundamental problems of Physics, namely, the “scattering problem”, where a particle (blue ball in Fig. 2a) is shot on a “target” (green ball), and its deviation from the straight line motion is used to study the interaction potential. In the original scattering problem, this deviation is assessed by accounting for the straight-line distance *b* (called the impact parameter) and closest approach $r_{\text {min}}$, which is derived from the scattering angle $\theta$, as an accurate measurement of particles’ location is very difficult. By repeating the experiment with different impact parameters and estimating the corresponding closest approach $r_{\text {min}}$, one can get an approximation for the potential acting on the particle.

In this study, we establish a simple duality relation between the above-mentioned problem and our group-individual collision avoidance scenario. Namely, the impact parameter *b* is replaced with a *straight-line distance* $r_b$ and the closest approach can simply be measured as the shortest distance $r_0$ between pairs of trajectory data points of the group and the individual. In section  “Observables” we elaborate in detail on how we define these observable quantities.

Using this approach from physical sciences to describe human behavior represents obviously a strong approximation, not only because human behavior is too complex to be modeled through simple physical forces, but also because it completely ignores the effect of the environment, which, in physical parlance, is equivalent to a strong and non-uniform external force. Nevertheless, as we will see, this approach still allows us to grasp the fundamentals of the collision dynamics between groups and individuals and to quantify the interaction. At this point, it is also worth stressing that the proposed model in section “Modeling” is aimed at assessing the effect of different social attributes in a qualitative way, rather than reproducing quantitatively human behavior.

### Observables

#### Data preparation and transformation

We first carry out a data preparation step by (i) removing atypical/non-characterizing motion (waiting, running etc.), (ii) representing the group as a single unit (its geometrical center) and (iii) focusing on frontal encounters of groups and individuals, for which we expect the pedestrians to be able to judge the social attributes of the incoming party.

Subsequently, we transform trajectories of the group *g* and the individual *i* to a reference frame, which is co-moving with the group. Namely, at each time instant (i) the positions of the group and the individual ${\textbf{r}}_{g,i}$ are translated such that the group (center of mass) is positioned at the origin and (ii) their velocities ${\textbf{v}}_{g,i}$ are rotated such that the velocity of the group is directed towards $x^+$. Finally, the velocities ${\textbf{v}}_{g,i}$ are translated by $-{\textbf{v}}_{g}$, rendering the group immobile. The main purpose of this transformation is to provide an easier visualization of relative position in 2D, which represents the position of the individual with respect to the group center, while having the group motion as a preferential direction. On the other hand, most of our analysis is based on the absolute value of the relative distance between the group center and the individual, which is rotationally invariant and independent of frame choice.

#### Relative distance r

Our analysis is based on ${\textbf{r}}$, the relative position between the group center and the individual,

1 $$\begin{aligned} {\textbf{r}}(t)={\textbf{r}}_{i}(t)-{\textbf{r}}_{g}(t). \end{aligned}$$

Its time derivative is the relative velocity ${\textbf{v}}$,

2 $$\begin{aligned} {\textbf{v}}(t)={\textbf{v}}_{i}(t)-{\textbf{v}}_{g}(t). \end{aligned}$$

The absolute value (norm) of ${\textbf{r}}$ is simply denoted as *r*.

#### Straight-line distance $r_b$

The straight-line distance $r_b$ is computed as the shortest distance from the origin (i.e. translated position of the group) to the line, which passes through the point at which the individual enters a pre-defined vicinity around the group termed as *window of observation*. This refers to the area in the group-centered reference frame from $-W$ to *W* meters both along *x* and *y* axes (i.e. along the group’s motion direction and the direction orthogonal to that). Empirically, a *W* of 4 m is seen to contain the most significant part of the group-individual collision avoidance (see Fig. 2b for an illustration. Refer to literature^53,54^ and Supplementary Information Section 3 for details on the choice of *W*).

Let $t^{\prime }$ be the time instant at which the individual enters *the window of observation* and let ${\textbf{r}}(t^{\prime })$ be its relative position at that instant. According to the hypothesis mentioned in section “Methods”, provided that there is no collision avoidance the individual will follow a path starting at ${\textbf{r}}(t^{\prime })$ and move along its velocity vector at that instant ${\textbf{v}}(t^{\prime })$. In this case, the straight-line distance $r_b$ can be computed as the shortest distance between this line and the origin (i.e. translated position of the group),

3 $$\begin{aligned} r_b=\frac{||{\textbf{r}}(t^{\prime })\times {\textbf{v}}(t^{\prime })||}{|| {\textbf{v}}(t^{\prime })||}. \end{aligned}$$

In the analysis, in order to alleviate the impact of orientation noise on the velocity of the individual, we averaged its velocity vector over four time instants (before $t^{\prime }$) and used this mean velocity in Eq. (3) instead of ${\textbf{v}}(t^{\prime })$.

#### Observed minimum distance $r_0$

The observed (i.e. actual) minimum distance $r_0$ between the group and the individual is simply,

4 $$\begin{aligned} r_0=\min _t\big (r(t)\big )= r(t_c), \end{aligned}$$

where $t_c$ is the time instant at which the individual is closest to the group. The times at which pedestrian positions are recorded, are obviously discrete. Nevertheless, in order to have a more accurate estimation of $r_0$, one can also interpolate ${\textbf{r}}(t)$ between two consecutive times $t_k$ and $t_{k+1}$ by using the velocity vector at time $t_k$,

5 $$\begin{aligned} {\textbf{r}}(t)\approx {\textbf{r}}(t_k)+(t-t_k){\textbf{v}}(t_k),\;t\in [t_k,t_{k+1}). \end{aligned}$$

This procedure allows detecting minimum distances not only exactly at sampling instants, but also at intermediate time points between consecutive samples, which yields a much more accurate estimation of $r_0$ (refer to Supplementary Information Section 4 for details).

#### Scaled distances

Groups’ interpersonal distance is shown to depend on their social relation and interaction intensity^17,18^. Thus, we represent the distances defined above in two ways: in a group-independent way (in meters) and in a group-dependent way, in which the unit of distance is the average interpersonal distance of dyads with the given social bonding^16^). In the text, we denote distances measured in meters with the normal font (e.g. *r*) and scaled distances measured in interpersonal distance units with a bar (e.g. ${\bar{r}}$). Since we observed that results concerning scaled values are in general easier to interpret, in the main text we mostly report those (for further details, refer to Supplementary Information Section 5).

### Analysis of collision avoidance

As mentioned in section “Methods”, our study of the collision avoidance dynamics between groups and individuals is fundamentally based on examining the relation between $r_b$ and $r_0$. To that end, in what follows we define two different methods to analyze this relation and then propose a method to model it.

#### Empirical relation between $r_b$ and $r_0$ and its statistical analysis

To examine the distribution of $r_b$ versus $r_0$, the values of ${\bar{r}}_b$ are quantized into bins of 0.5 unit and for each bin, the average and standard error of the corresponding values of ${\bar{r}}_0$ are computed. The choice of 0.5 as bin size was primarily driven by empirical observations. For certain combinations of distance $r_b$ (or ${\bar{r}}_b$) and bonding (social relation or interaction level), setting a smaller bin size results in having bins with little or no data. Conversely, using a larger bin size decreases the resolution. In that respect, we consider a bin size of 0.5 to strike a balance between these competing factors. The results will be presented and discussed in section  “Results” on the relation between r$_0$ and r$_{\rm b}$.

#### Intrusions

Small groups, such as dyads, have been shown to usually prefer deviating to avoid splitting^55^. Nonetheless, we found situations where the individual passes through the group (i.e. between group members), and we refer to them as “intrusion”. For simplicity’s sake, we define the probability of intrusion as the probability of having $r_0$ smaller than the group interpersonal distance (see section “Scaled distances”). We perform a statistical analysis to investigate the dependence of intrusion on the social attributes of the group. The results will be shown and discussed in section “Intrusion”, whereas the details of the computational procedure can be found in Supplementary Information Section 6.

#### Modeling

Many models of pedestrian collision avoidance are based on Social Forces^43–45^, which may be defined through a potential. It has been reported that using position-dependent potentials in modeling of pedestrian collision avoidance fails to reproduce detailed behavior. Even if we assume that a Social Force approach may reproduce actual human behavior, the corresponding potential should at least be velocity-dependent and based not on current distance but on future distance at the moment of predicted closest approach^13,45^. Nevertheless, determining a potential that may, at least qualitatively, describe the collision avoidance between groups and individuals, represents an important first step towards a more realistic quantitative modeling.

Let us first review how we can study the potential energy between two interacting bodies in physics (note that while discussing the physical model, we use the word “interaction” to refer to the effect that the bodies exert on each other.). The study of such a scattering problem is obviously a cornerstone of physics, and the non-Quantum formalism analyzed in this section was used to study such important problems as the structure of atoms^56^ and gravitational lens effect due to space-time curvature^57^ among others. In general, the bodies in focus are very complex and composed of many particles (e.g. planets, stars). Nevertheless, due to the scale of the problem, they may be treated as point particles themselves (in our “pedestrian scenario”, the group will be represented with a single point).

Their interaction is determined by a potential energy $U({\textbf{r}})$, which is in general a function of only relative position (a result connected to invariance under space translations and equivalent to Newton’s third law^58^). Nevertheless, in many important applications, the potential is central, i.e. rotationally invariant, and depends only on the magnitude of the distance, *U*(*r*).

In such a case, it is shown that the interesting (potential-dependent) dynamics is studied in the *r* variable^56^. Defining the reduced mass $\mu$ as

6 $$\begin{aligned} \mu =\frac{m_1 m_2}{m_1+m_2}, \end{aligned}$$

where $m_1$ and $m_2$ are the masses of the two bodies, the angular momentum ${\textbf{L}}$ and energy *E* result to be constants of motion,

7 $$\begin{aligned} E=\frac{\mu }{2}{\dot{r}}^2+\frac{||{\textbf{L}}||^2}{2\mu r^2}+U(r). \end{aligned}$$

In a scattering problem, the system is not bound and *r* diverges for $t\rightarrow \pm \infty$. In most physical applications, we can only measure the scattering angle and the velocity far before/after the interaction. We can then compare the measured angle with a theoretical result involving an integral. However, if the full trajectory is known, a simpler way to study the system is available.

We study the system far before interaction, i.e. for $t\rightarrow -\infty$ and $r \rightarrow +\infty$, and call the corresponding asymptotic speed $v_{\infty }$. We see that the absolute value of angular momentum can be written as

8 $$\begin{aligned} L\equiv ||{\textbf{L}}||=\mu v_{\infty } b, \end{aligned}$$

where the impact parameter *b* is the minimum value of *r* assumed in case of straight motion (i.e. no interaction). Assuming $\lim \limits _{r\rightarrow +\infty } U(r)=0$, we obtain

9 $$\begin{aligned} E_{\infty }=\frac{\mu }{2}v_{\infty }^2. \end{aligned}$$

On the other hand, since we actually have interaction, the minimum distance $r_{\text {min}}$ in the observed trajectory turns out to be different than *b*, i.e. $r_{\text {min}}\ne b$. At $r=r_{\text {min}}$, having a minimum, we have ${\dot{r}}=0$ and the corresponding energy is

10 $$\begin{aligned} E_0=\frac{\mu v_{\infty }^2 b^2}{2 r_{\text {min}}^2}+U(r_{\text {min}}). \end{aligned}$$

Conservation of energy implies $E_{\infty }=E_0$ and provides the following relation for the value of *U*(*r*) at $r=r_{\text {min}}$

11 $$\begin{aligned} U(r=r_{\text {min}})=\frac{\mu v_{\infty }^2}{2}\frac{r_{\text {min}}^2-b^2}{r_{\text {min}}^2}. \end{aligned}$$

This relation enables studying the potential *U*(*r*), provided that $v_{\infty }$, *b* and $r_{\text {min}}$ are measured. In modeling collision avoidance between pedestrians based on the above framework, we assume that

12 $$\begin{aligned} \frac{\textrm{d}U(r)}{\textrm{d}r}<0\; \forall r \Rightarrow U(r)>0 \; \forall r. \end{aligned}$$

In other words, the force is assumed to be repulsive. Namely, denoting ${\textbf{F}}_1$ as the force acting on body 1, and recalling the usual definition ${\textbf{r}}={\textbf{r}}_1-{\textbf{r}}_2$, we have

13 $$\begin{aligned} {\textbf{F}}_1=-\varvec{\nabla }U(r)=-\frac{\textrm{d}U(r)}{\textrm{d}r}\frac{{\textbf{r}}}{r}. \end{aligned}$$

We apply these physical concepts in a pedestrian scenario to model the “collision avoidance potential” between groups and individuals. As mentioned in section “Methods”, $r_b$ is inspired by the impact parameter *b*, whereas $r_0$ corresponds to the closest approach $r_{\text {min}}$. Thus, the term $v_{\infty }$ in Eq. (11) should be approximated by using the relative velocity when $r_b$ is computed. But since pedestrian velocities have a small variation, we may consider it to be almost constant. In a similar way, as usual when studying “forces” that determine the pedestrians’ cognitive decisions, all masses are considered to be equal (to one)^43^ and we may remove $\mu$ from the equation. Finally, since the approach is completely of a qualitative nature, we opt for ignoring the overall constant in Eq. (11) and study the following simplified version,

14 $$\begin{aligned} U^\prime (r=r_0)=\frac{r_0^2-r_b^2}{r_0^2}, \end{aligned}$$

to which we will refer to as the “collision avoidance potential” (defined as a dimensionless pure number).

A comment on Eq. (14) is probably needed. This equation does not represent the functional form of the dependence of the potential on *r*. Instead, it shows which is the value of *U* attained at $r_0$ given that the straight-line distance is $r_b$. Different values of $r_b$ allow us to probe different values of *U*, where the smaller *b* is, the higher $U'$ is. Nevertheless, Eq. (14) clearly allows us only to probe values $U'<1$. This is due to the fact that in the computation of $U'$ the value of the initial kinetic energy

15 $$\begin{aligned} \frac{\mu v_{\infty }^2}{2} \end{aligned}$$

is taken as fixed, and we are measuring the probed values of the collision avoidance potential as multiples of such kinetic energy. Note that in particle physics short distances are indeed probed by using very high kinetic energies.

The results are shown and discussed in section “Potential”, whereas the details of the computational procedure are described in Supplementary Information Section 7. In addition, in section  “Comparison to individual–individual collision avoidance” we also show the results concerning a similarly defined potential describing individual–individual collision avoidance.

## Results and discussion

### Results on relative frame pdfs

**Figure 3:**
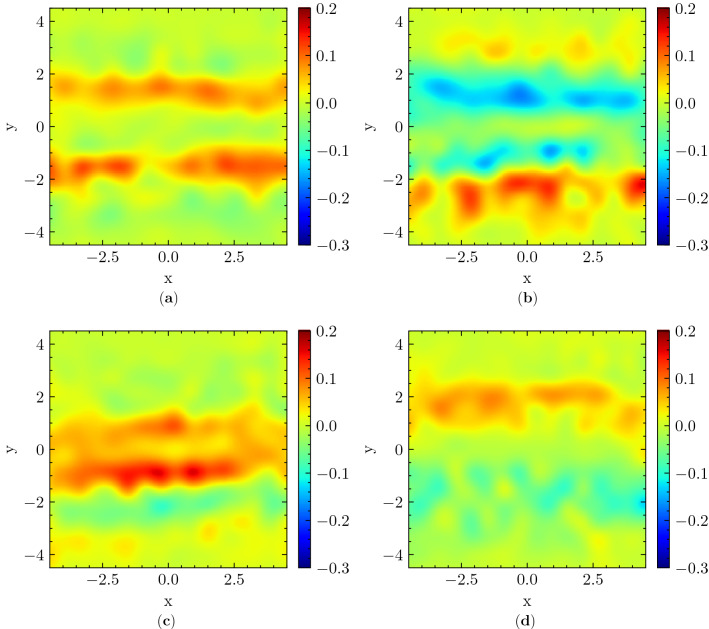
2D probability distribution of individuals’ position $\bar{{\textbf{r}}}$ relative to overall average. Positions are shown in the group-centered reference frame and the *x* axis is aligned with the direction of motion of the group. Each sub-figure depicts the difference between the distribution relating to a certain social relation and an unweighted average concerning all social relations. (**a**) Colleagues, (**b**) couples, (**c**) families, (**d**) friends. The color scales are adjusted for highlighting the differences.

**Figure 4:**
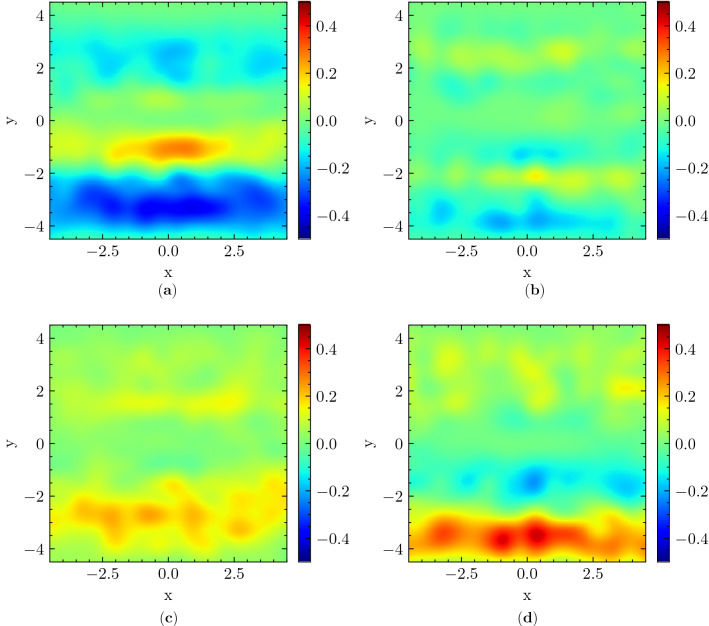
2D distribution of individuals’ position $\bar{{\textbf{r}}}$ relative to overall average. Positions are shown in the group-centered reference frame and the *x* axis is aligned with the direction of motion of the group. Each sub-figure depicts the difference between the distribution relating to a certain intensity of interaction and an unweighted average of all intensities. (**a**) 0, (**b**) 1, (**c**) 2, (**d**) 3. The color scales are adjusted for highlighting the differences.

The *group-centered reference frame* is particularly suitable to study the 2D distribution of ${\textbf{r}}$, i.e. of the position of the individual around the group. Figures 3 and 4 show the 2D distributions in relation to different social relations and interaction intensities of the group, respectively, using as a distance unit the groups’ average interpersonal distance. Note that, in order to highlight the specificities of each social attribute as compared to the whole, we depict the difference between a given attribute and the overall 2D average, which is computed as an unweighted average of the distributions of all relating cases. Therefore, positive values depict an increased likeliness of presence for the individual, while, reciprocally, negative values depict a decreased likeliness.

The effects of varying social relations are presented in Fig. 3. Comparing Fig. 3a with b, d, one may notice that individuals do not have a prominent preference to pass on the right or left side of colleagues, whereas they prefer to pass more on the right for couples and on the left for friends (as compared to the overall average). In addition, they pass with a very small distance ($r \approx 0$) more often for families (see Fig. 3c) than for other kinds of social relations, which may be due to a more dispersed configuration of family group members^18^. On the other hand, in Fig. 3b we see very clearly two low probability horizontal stripes, roughly located around $y=\pm 1$. As these stripes correspond more or less to group members’ positions, they suggest that the group’s abreast formation is rarely disturbed in couples.

Concerning social interaction, the difference with respect to varying intensities is much more noticeable, the most interesting one being between 0 and 3 (see Fig. 4a,d). Namely, concerning groups annotated as non-interacting (i.e. with 0 intensity of interaction), the center stripe presents positive values, while the lower and upper stripes $y \approx \pm 2.5$ present negative values, indicating that individuals are more likely to maintain a trajectory directly facing the group (possibly even intruding it) (see Fig. 4a). Reciprocally, from Fig. 4d we can see that individuals are less likely to position themselves on a colliding trajectory with the group and prefer to place themselves on its side, when it has a high intensity of interaction. There are interesting left/right asymmetries in Fig. 4, which may be related to the tendency of Japanese pedestrians to move mainly on the left, and overtake on the right^59^. This tendency may cause low-interaction groups, when they are not intruded on, to have a relatively higher possibility to be passed on their left than on their right, since they are expected to have a higher speed than highly interacting ones. We do not have a clear interpretation for the right/left asymmetry between the couples distribution in Fig. 3b and the friends distribution in Fig. 3d.

### Results on the relation between ${\bar{r}}_0$ and ${\bar{r}}_b$

**Figure 5:**
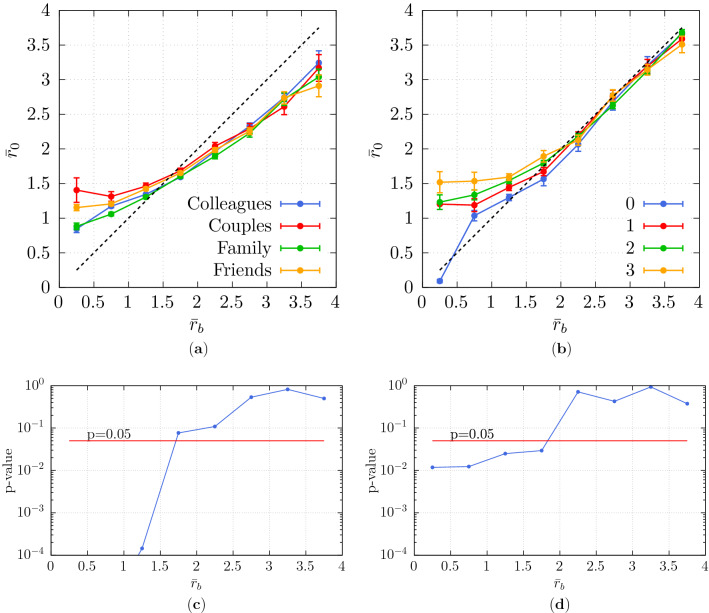
Observed minimum distance ${\bar{r}}_0$ as a function of the undisturbed straight-line distance ${\bar{r}}_b$ (**a**) for various social relations and (**b**) intensities of interaction of the group. Error bars report standard error intervals. The dashed line corresponds to the ${\bar{r}}_0={\bar{r}}_b$ linear dependence. *p*-values for the ANOVA of ${\bar{r}}_0$ (**c**) for various social relations and (d) intensities of interaction of the dyad. In (**c**), results for ${\bar{r}}_b<1$ are not displayed as very low values were obtained ($p < 10^{-6}$).

We divide the range of ${\bar{r}}_b$ into bins of 0.5 unit and compute the mean and standard error of ${\bar{r}}_0$ corresponding to each bin. The results are depicted in Fig. 5a,b. Smaller values of ${\bar{r}}_b$ indicate that the straight line trajectory of the individuals would require them to pass very close to the group. In addition, ${\bar{r}}_b < 0.5$ signifies a distance smaller than half of the group interpersonal distance, which means that the individual would need to *intrude on the group* (if moving straight).

Concerning social relations, we observe that when ${\bar{r}}_b < 0.5$, the average value of ${\bar{r}}_0$ is considerably larger for couples and friends than for colleagues and families (see Fig. 5a). In other words, there is a strong resistance against intruding on groups with the former social relations. Concerning intensity of interaction, we have a similar observation for higher intensities of interaction (from 1 to 3, see Fig. 5b), while for 0 intensity we have ${\bar{r}}_0\approx {\bar{r}}_b$ for all ${\bar{r}}_b$ values.

These observations make us believe that the social attributes of the group do impact group-individual collision avoidance. Specifically, there is larger avoidance, when there is a strongly-bonded group involved (i.e. couples, friends or with high intensity of interaction).

The statistical significance of these results can be assessed through an ANOVA (see Supplementary Information Section 8 for considerations regarding the necessary assumptions). To that end, we compute the *p* values concerning each bin shown in Fig. 5a,b and demonstrate the results in Fig.  5c,d, respectively. Regarding lower values of ${\bar{r}}_b$ (i.e. ${\bar{r}}_b < 1.5$), we observe statistical significance (i.e. $p < 0.05$) concerning both social relation and intensity of interaction. Regarding larger values of ${\bar{r}}_b$ (i.e. ${\bar{r}}_b > 2$), there is no statistically significant difference, as it can be expected observing the overlapping curves in the corresponding regions of Fig. 5a,b.

Let us also notice that in Fig. 5b concerning the DIAMOR data set, for ${\bar{r}}_b \gg 1$, we have ${\bar{r}}_b \approx {\bar{r}}_0$ regardless of intensity of interaction, in agreement with the hypothesis that collision avoidance can be ignored for such values (Eq. (14)). The fact that this is not the case in ATC, where we actually observe ${\bar{r}}_b > {\bar{r}}_0$ for ${\bar{r}}_b \gg 1$, is considered to be an effect of the ATC environment being less straight and narrower (see Fig. 1a).

To compensate for this effect in the computation of the potential, we perform a linear correction in the computation of ${\bar{r}}_b$ in section “Potential”. The details of this correction are presented in Supplementary Information Section 7. In addition, results concerning the relation between $r_b$ and $r_0$, i.e. values measured in meters and not scaled with group interpersonal distance, are shown in section “Comparison to individual–individual collision avoidance”.

### Intrusion

It is noticeable that the observed minimum distance ${\bar{r}}_0$ reaches particularly low values in some encounters. For instance, the first bin in Fig. 5b for intensity of interaction 0 presents an average value of ${\bar{r}}_0$ smaller than 1. This means that the distance from the center of mass of the group to the individual gets smaller than the group interpersonal distance (see Supplementary Information Section 5). In such cases, it is likely that the individual is actually intruding on the group instead of deviating, essentially following the straight line trajectory.

**Figure 6:**
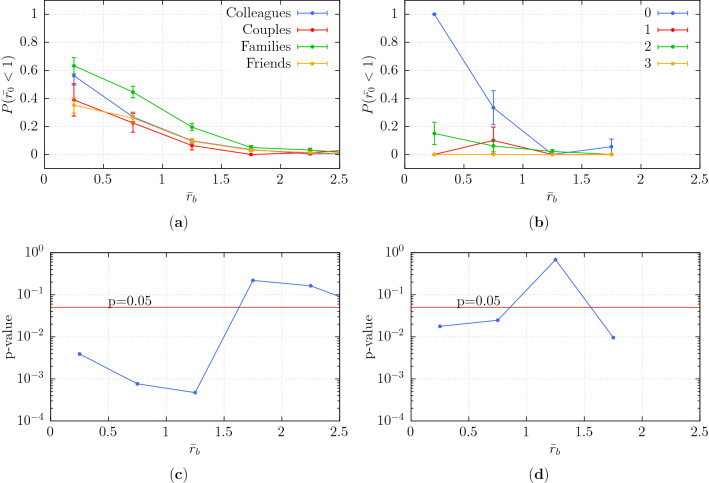
Probability that the distance ${\bar{r}}_0$ is smaller than 1 for (**a**) for various social relations and (**b**) intensities of interaction of the dyad. Pearson’s $\chi ^2$ $p$-values for the hypothesis of independence of the frequencies of samples verifying ${\bar{r}}_0 < 1$ for (**c**) for various social relations and (**d**) intensities of interaction (of the group).

To quantify the frequency of such intrusions, we computed the probability of ${\bar{r}}_0$ being smaller than 1. Specifically, this is an empirical probability computed as the ratio of the number of observations with ${\bar{r}}_0 < 1$ to the total number of observations (for a given bin of ${\bar{r}}_b$). The results are shown in Fig. 6a,b. Here, we see that there is indeed a correlation between the probability of intrusion and the social bonding of the group being intruded on. Namely, individuals have a higher probability to intrude on loosely-bonded groups (i.e. colleagues, families and non- or slightly-interacting groups) than strongly-bonded groups (couples, friends and strongly interacting groups).

The statistical significance of this observation is assessed through Pearson’s $\chi ^2$ test and the relating *p*-values are presented in Fig. 6c,d. The difference in probability of intrusion concerning different social relations is significant ($p < 0.05$), when ${\bar{r}}_b$ is smaller than 1.5. On the other hand, for the intensity of interaction we have a significant difference of intrusion for ${\bar{r}}_b < 1$.

Actually, the average distance of a group member from the group center is ${\bar{r}}_0=0.5$. The corresponding analysis for the probability of having ${\bar{r}}_0<0.5$ is shown in Supplementary Information Section 6.

### Potential

**Figure 7:**
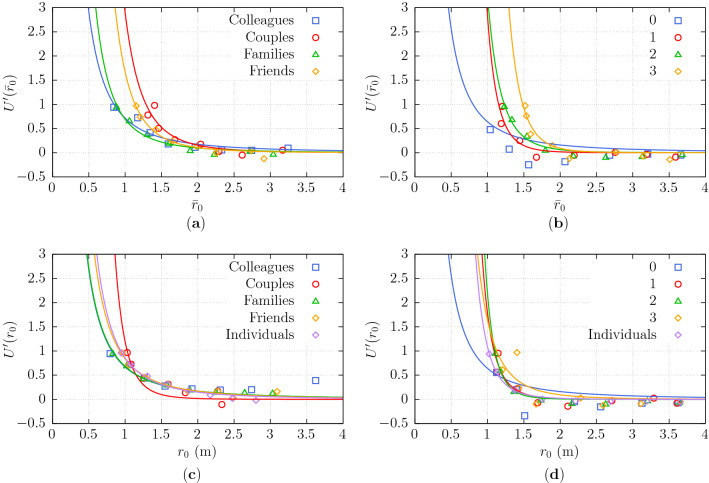
Collision avoidance potential $U^\prime ({\overline{r}}_0)$ (**a**) for various social relations and (**b**) intensities of interaction of the group. Dashed lines correspond to a power function fit of the quantized data. Collision avoidance potential $U^\prime (r_0)$ (**c**) for various social relations and (**d**) intensities of interaction of the group. Dashed lines correspond to an exponential fit of the quantized data. (**c,d**) report a comparison to individual-individual (non-group) interaction using non-scaled distances.

As described in section “Modeling”, we study $U^\prime (r_0)$ (see Eq. (14)) to model the “potential” representing the interaction between the group and the individual. To that end, we again quantize the values of ${\bar{r}}_b$ and compute the corresponding mean values of ${\bar{r}}_0$ before calculating the values of the potential $U'({\bar{r}}_0)$ for each bin. Figure 7a,b show the relating values. Additionally, to extrapolate outside the range available, a function of the form ${k}/{r^\beta }$ is fitted to the data using non-linear least squares, illustrated with dashed lines in Fig. 7a,b.

Interestingly, the potential is shown to be affected by the nature of the social bonding of the group. As a matter of fact, stronger bondings (e.g. couples, high intensity of interaction) generate a “stronger potential” (i.e. with a steeper negative derivative) which, as seen in sections “[Sec Sec17]” and “Intrusion” “causes” individuals to deviate more, and significantly decreases their probability to intrude on the group. On the other hand, loosely-bonded groups (e.g. colleagues and non- or slightly-interacting groups) generate a weaker potential, resulting in a smaller deviation and a higher chance of intrusion.

The discussion above concerns results obtained using distances scaled with the group interpersonal distance; results concerning computations performed using distances measured in meters are shown in section “Comparison to individual–individual collision avoidance”.

### Comparison to individual–individual collision avoidance

Many practitioners simulate crowds on the basis of individuals. Thus, it is interesting to compare the above-mentioned potentials with results obtained for individual–individual interaction. The results (using distances measured in meters, i.e. not scaled by group interpersonal distance) are shown in Fig. 7c,d.

Note that groups are larger (than individuals) and expected to exert a stronger “social force”, but they are also susceptible to being disrupted and intruded on (passed at $\approx 0$ distance to their geometrical center). Also, while it is expected (statistically) that collision avoidance between individuals is symmetric, it may be that groups interact less than individuals by deviating very little. These effects seem to balance and potentials for collision avoidance between individuals are quite similar to those with groups.

Nevertheless, it may be seen that potentials describing low intensity social interactions, colleagues and families have typically a less steep derivative than the one for individual-individual encounters, while the opposite is observed for high-intensity social interactions and (in particular) for couples.

## Conclusion

In this work, we analyzed how group-individual pedestrian collision avoidance depends on the group’s social relation and social interaction intensity.

In detail, we verified that when straight motion (i.e. absence of collision avoidance) would lead to a possible collision, the actual minimum distance $r_0$ between the individual and the group is a growing function of social interaction intensity, and assumes a higher value for couples and friends. Similarly, individuals have a stronger tendency to intrude or disrupt a group by passing at a distance comparable to the group interpersonal distance when they face groups with low interaction intensity and colleagues and families, as can be verified both by studying 2D distance probability distributions, and by performing a statistical analysis on the probability that the minimum distance becomes smaller than the group interpersonal distance.

We also introduced a potential to study the dependence of intensity of collision avoidance on relative distance, by mimicking the theoretical modeling of two-body scattering in classical mechanics. This approach, which may be used as a guiding light in the development of a social force model of individual–group interaction, shows again that the potential determining collision avoidance tends to grow much faster with decreasing distance values (i.e. it has a steeper negative derivative) for strongly interacting groups, couples and families.

The latter result is particularly clear when studied using the group’s average interpersonal distance as a length unit. A further comment on this result may be necessary, since the tendency of individuals not to pass through strongly bonded dyads (such as couples, friends and strongly interacting dyads) may be due not only to some kind of social rule, but also to the fact that passing through these groups is actually harder due to the narrower space between them.

To this respect, we should finally comment also on the results concerning families, which may be a little counter-intuitive by suggesting that families are somehow perceived as weakly interacting and are often “intruded”^60^. It should be stressed that, as reported by Zanlungo et al.^16^, the families in the ATC data set are mostly composed of parent-child pairs, that often do not walk abreast, or at least have a weaker tendency to walk abreast. The authors of the original study justify this tendency by referring to “the erratic behavior of children”, but it may also be related to a stronger hierarchical structure in a parent-child dyad with respect to couples, friends and colleagues^16^. It may thus be argued that the tendency of individuals to approach families at a shorter distance may depend on families being less spatially structured, or correspondingly having a higher tendency to change their spatial structure. Such role of group spatial structure in individual-group interaction could be the subject of future studies, possibly when larger data sets collected in more suitable environments will be available.

We believe that our results and inferences point out interesting variabilities in pedestrian motion due to social aspects of human navigation^61^. A valuable implication of our study is that infrastructure design could be adapted to the nature of the social bonding of its users. We can speculate that, for instance, if a particular environment is known to be frequented mostly by strongly bonded groups, such as an amusement park, providing additional space (e.g. by widening corridors or walkways) to allow for collision avoidance may make it more comfortable. Nevertheless, these qualitative considerations should ultimately be corroborated with quantitative simulation models that include our findings. By taking into account the social dynamics of the people using a particular space, designers and architects could create environments that are more conducive to safe and efficient movement. This could help to reduce the risk of accidents and improve overall user experience. We also hope that using models which account for the expected social composition of the crowd may help in improving the performance of tracking and simulation systems^62^.

## Supplementary Information

Supplementary Information.

## Data Availability

The data sets analyzed during the current study are available at https://dil.atr.jp/ISL/sets/groups/.
