# Supplementary material for: Social aspects of collision avoidance: a detailed analysis of two-person groups and individual pedestrians
Source: Sci Rep. 2023 Apr 8;13:5756. doi: 10.1038/s41598-023-32883-z (PMC10082808; doi:10.1038/s41598-023-32883-z)
Supplement: Supplementary file 1 — Supplementary Information. [file 41598_2023_32883_MOESM1_ESM.pdf]

Supplementary information for the article
Social aspects of collision avoidance: A detailed analysis of two-person groups and individual pedestrians

Adrien Gregorj[1], Zeynep Yücel[1,3], Francesco Zanlungo[1,2,3], Claudio Feliciani[4], Takayuki Kanda[3,5]

1 Okayama University, Okayama, Japan
2 Osaka International Professional University, Osaka, Japan
3 ATR International, Kyoto, Japan
4 The University of Tokyo, Tokyo, Japan
5 Kyoto University, Kyoto, Japan

1 Specifics of experiments and data

In this section, we give details about experimental conditions and specifics of data and annotations.

ATC data set

The ATC data set is a large pedestrian data set, which is introduced by Zanlungo et al.[1] and has since been used in several studies, especially those focusing on social aspects of crowd dynamics[2–7] and it is freely available[8]. It has several properties, which make it particularly fit for the investigation of such factors and we will point them out as we explain the specifics of the data set below.

The recording location is the ground floor of a multi-purpose center, which involves offices, shops and facilities for special events such as expositions. In particular, the tracking area consisted of an atrium and a corridor which, along with being bordered by shops, serve also as a passage between a train station, a ferry terminal and the office area. Therefore, in addition to accommodating the visitors of the multi-purpose center, it is also constantly frequented by commuters. In that respect, the recorded pedestrians come from a diverse social background (e.g. age group, occupation etc).

The recording area is quite large, i.e. approximately 900 m^2. Therefore, it allows continuous tracking of individuals for long distances (i.e. up to 50 m), which helps also in the annotation of social relations. Note that in the analysis we discarded the trajectory segments collected in the atrium and used the segments collected in the corridor illustrated in Figure 1-(a), which is over 40 m long.

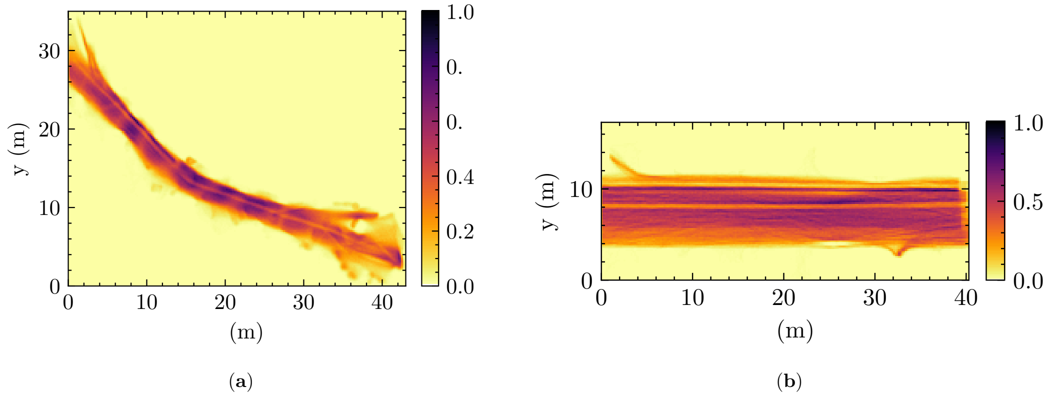

Figure 1. The normalized cumulative density maps for (a) the ATC data set and (b) DIAMOR data set.

The recording time span is more than 800 hours within a one year time window. In such, it involves workdays, weekends, national holidays, public festivals, and special days of the business center such as exhibitions and trade shows. These occasions lead to an increase in density and a diversity in the purpose of visit.

The raw data are in specific depth and video information registered by an RGBD sensor network. Based on the depth information and the algorithm of Brščić et al.[9], the pedestrians are tracked automatically (on the 2D floor plane) and the resulting trajectories can freely be downloaded[8]. The concerning normalized cumulative density map of the environment is

found as in Figure 1-(a). Note that the cumulative density map is basically a 2D histogram. We consider the environment as a 2D mesh with a grid cell size of 10 cm by 10 cm and count the number of observations in each grid cell. The normalization refers to the scaling of this histogram with its maximum value.

Note that since the sampling frequency of the sensors is quite high (i.e. 20 Hz), tracked trajectories are influenced by gait dynamics. Furthermore, if velocities were simply computed as discrete differences between the tracked trajectory points, the relatively small error in position due to sensor noise would be magnified. For this reason, Zanlungo et al. proposed re-sampling the data by averaging pedestrian positions over 0.5 s time windows, in order to reduce the influence of pedestrian gait and other sorts of measurement noise, as well as to assure uniform sampling in time, and also in this work we are going to use such re-sampled data[1].

Based on the video information, the pedestrians are annotated according to group membership and several intrinsic group features[10]. Specifically, human coders labeled which pedestrians constitute a social group and which pedestrians move alone. In addition, for the groups they also annotated intrinsic features such as age, purpose of visit etc. One specific feature, which we focus on in this study, refers to the apparent social relation of the group members. The possible options are couples, colleagues, family and friends, which correspond to the domains of mating, coalitional, attachment and reciprocal defined by Bugental[11], respectively. (See Table 1-(a) for the outcome of the annotation process).

Table 1. Number of groups annotated with each (a) social relation (in ATC data set) and (b) intensity of interaction (in DIAMOR data set).

(a)

Social relation	# of annotations
Couples	69
Colleagues	314
Family	180
Friends	253
Total	817

(b)

Intensity of interaction	# of annotations
0 (no interaction)	140
1	159
2	460
3 (strong interaction)	100
Total	859

DIAMOR data set

The DIAMOR data set was introduced by Zanlungo et al.[12] and used particularly for the purposes of group recognition and motion modeling[12–14].

The recording location is an underground pedestrian street network in a commercial district of Osaka, Japan. The entire underground network is composed of a total of more than several kilometers of walking path, and it is connected to the Osaka-Umeda railway and underground station complex, which is considered to be one of the busiest in the world (the busiest outside Tokyo) and visited daily by millions of pedestrians. For example, according to the Osaka municipal transportation bureau, the three metro stations located in the underground area had a daily number of passengers of more than 700K in the fiscal year 2019. The DIAMOR data set includes recordings from a junction of two straight corridors in a relatively peripheral portion of this street network and we focus on one of these. Similar to ATC data set, several train stations, business centers, shopping malls etc. are accessible from the recording location leading to diversity in pedestrian profile.

The recording area is roughly 200 m^2 and allows continuous tracking along approximately 50 m. The recording time span is two weekdays and a total of eight hours of recordings are available, which, although shorter than the ATC data set, we consider to be enough for the purposes of this study.

Similar to the ATC data set, it is composed of depth and video information. The depth information is used to derive the trajectories of the pedestrians based on the method reported by Glas et al.[15] and the trajectories can be freely downloaded[8]. As a result of this tracking process, the normalized cumulative density map shown in Figure 1-(b) is obtained.

Based on the video information, human coders were asked to annotate groups and individuals (people who do not belong to a group). Of course, it is not wrong to say that each group member is an "individual", but within the context of this study we refer to them explicitly as "group member", and use the word "individual" specifically to refer to people who do not belong a group. Coders also annotated whether or not members of dyads were engaged in interaction (oral communication, possibly accompanied with non-verbal elements such as gestures or gaze exchange, as defined by Knapp et al.[16]), and the corresponding intensity of interaction (evaluated at 4 degrees from 0 representing no-interaction to 3 representing strong-interaction). Note that the annotations of ATC data set are inherently disjoint, i.e. the annotation labels can be considered to be mutually exclusive nominal variables. On the other hand, the annotation labels of the DIAMOR data set can be viewed as ordinal variables (i.e.

with a gradual relation). The outcome of the annotation process (i.e. the number of observations for each intensity of interaction) is summarized in Table 1-(b).

2 Data preparation

Both the ATC data set and the DIAMOR data set are collected in an ecological environment. Namely, they contain trajectories collected from uninstructed people moving freely. Although they were not recorded secretly (i.e. there were signboards informing the pedestrians that a data collection campaign was being carried out), the pedestrians' awareness of being recorded is anticipated to have a negligible effect on how they move, in particular as compared to participant experiments performed in artificial (laboratory) environment. In that respect, since the data are collected under uncontrolled settings, the tracked trajectories may contain behaviors like waiting, running etc. From the point of view of this study, such cases are not of interest. In order to eliminate atypical/non-characterizing observations, each trajectory is treated as explained below.

Let a group (dyad) be described as an unordered pair composed of (two members) p and q, i.e. $g = (p,q)$ and let i denote an individual. For the sake of simplicity, we reduce a group to a single mobile agent in the data preparation phase. Namely, the location of the group is represented by the *group center of mass* $\mathbf{r}_g$ and its velocity is represented with *group velocity* $\mathbf{v}_g$. Specifically, concerning a group $g = (p,q)$, $\mathbf{r}_g$ and $\mathbf{v}_g$ are represented as the average positions and velocities of p and q at each time step, respectively.

$$\begin{aligned} \mathbf{r}_g &= \frac{\mathbf{r}_p + \mathbf{r}_q}{2}, \\ \mathbf{v}_g &= \frac{\mathbf{v}_p + \mathbf{v}_q}{2}. \end{aligned} \tag{1}$$

Thereby, we treat a group g and an individual i in the same manner and first check the sufficiency of the number of trajectory data points. Provided that $| \left\{ \mathbf{r}_{g,i} \right\} | \geq 16$, the trajectories are considered to have enough data points for characterizing locomotion. Note that this corresponds to a minimum of 8 seconds of observation, since the sampling time step is 0.5 s. Any trajectory which includes fewer samples is discarded.

Next, we check instantaneous speeds $v_{g,i}$ and remove the trajectories which are associated with too low or too high speeds (i.e. out of walking range). For judging the typical speed range of pedestrians, we referred to the literature on human locomotion. Based on the results reported by Zanlungo et al. [12], g and i are considered to depict typical walking motion, if their instantaneous speed lies within the range $0.5 \leq v_{g,i} \leq 3$ (in m/sec). Otherwise, they are assumed to be "not walking" and discarded.

As mentioned in Section Introduction of the main track, we focus on the effect of social attributes of (the group) on collision avoidance. To have an understanding of those, the peers need to have sufficient visual information about each other. Therefore, we start with conditioning on having *a frontal view of each other*, which implies moving in opposite directions. In other words, if g and i move in the same direction, one party will be leading and the other following, such that the leading party will not see and thus not be aware of the other, and the following party will have limited information about the leading one (e.g. on social relation, age, interactions, etc.). In theory, the group and individual might also approach each other at an angle, but in the studied environments such cases are rare and we do not consider them in this work. However, if they move in opposite directions, they will have the opportunity to watch the incoming party and get a sense of its social features.

For ensuring a frontal view, we detected the relative motion direction of g and i and considered only those g and i, which move in opposite directions. Let ϕ represent the angle between the velocity vectors $\mathbf{v}_g$ and $\mathbf{v}_i$ at a given time instant,

$$\phi = \arccos(\mathbf{v}_g \cdot \mathbf{v}_i)/(||\mathbf{v}_g|| \, ||\mathbf{v}_i||). \tag{2}$$

Then, g and i are considered to be moving in opposite directions, if $3\pi/4 \leq \phi < \pi$. Note that, in addition to boasting a bigger potential from the viewpoint of our purposes, in the studied "bi-directional" environments, considering opposite relative motion direction has also the advantage that the number of observations associated with it is significantly higher than those with other directions (namely, 68% of all observations), which is preferable for the statistical analysis performed in our study.

3 Window of observation

As measures at infinity are obviously not feasible, in order to measure the straight-line distance r_b we need to define a window of observation (centered on the group). In particular, this window should satisfy two competing properties, *completeness* and *atomicity*. On the one hand, the window needs to be large enough to verify that, when entering and exiting from the side of the window the individual is not yet (significantly) influenced by the group (i.e. he/she is not yet engaged in an avoidance maneuver). This guarantees that the deviation is entirely contained in the window of observation, hence the notion of *completeness*. On the other hand, the window should not be too large, so that the assumption that the individual would walk

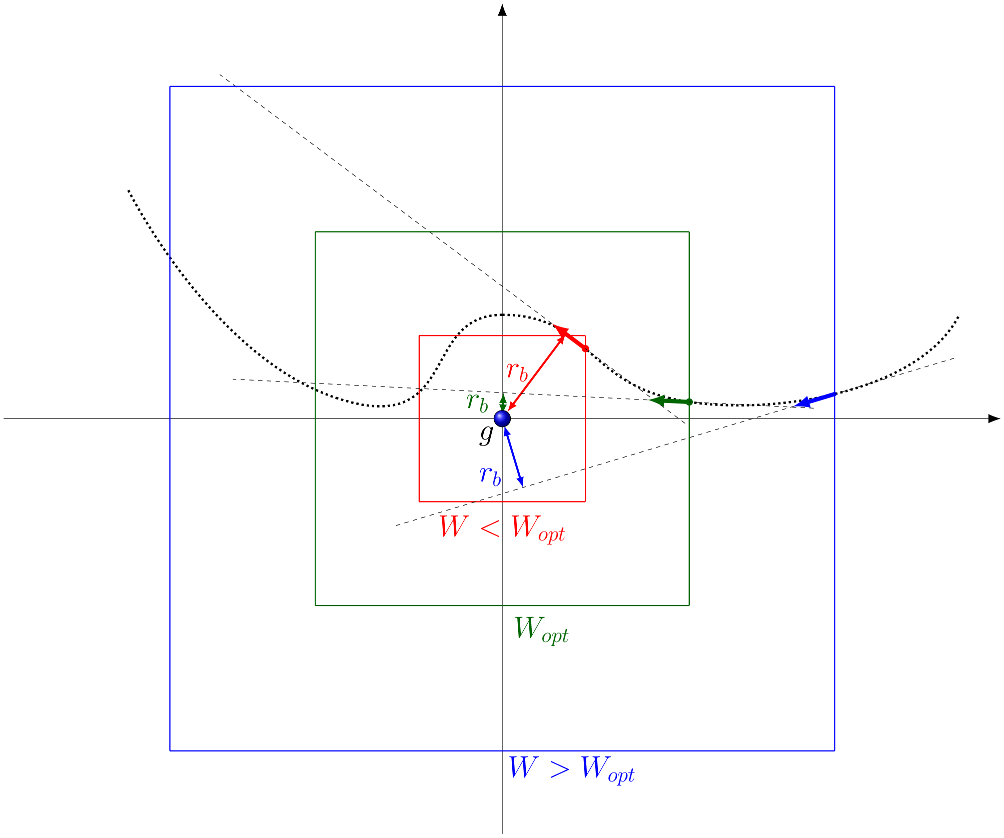

Figure 2. Illustration of the impact of the choice of the window size W on the computation of the straight-line distance r_b. In red, a small window violating *completeness*, in blue, a large window violating *atomicity* and in green, an "optimal" window which satisfies both constraints. Note that since we study frontal encounters in the group reference frame, individuals will always enter the window from the right.

on a straight line in the absence of the group stays valid. Indeed, although pedestrians certainly do not walk on straight lines at all times, at a relatively small scale and in the absence of perturbations (resulting from either external sources such as the environment or other pedestrians, or internal sources such as a change of planned destination) it can be expected that one's trajectory will be close to a straight line (*atomicity*). Figure 2 illustrates the impact of the size of the window.

Regarding *completeness*, we referred to literature on collision avoidance and searched for a reasonable threshold value. Cinelli and Patla found that, the "safety zone", i.e. the area in which individuals allow a moving object to approach before initiating an avoidance behavior, is on average 3.73 m[17]. Furthermore, Kitazawa et al. showed that pedestrians gaze most at other approaching individuals, when they are on average 3.97 m away, and that they seldom look at pedestrians at longer distances than this[18]. Therefore, we deliberated that 4 m is a reasonable lower bound for considering that their mutual influence is still null.

Regarding *atomicity*, the environment needs to be taken into consideration. In a straight and wide corridor, like in DIAMOR, the straight line assumption is more founded than for a more complex environment, like the bent and narrow corridor in ATC where pedestrian have to follow the curve and will have naturally less straight trajectory. Nonetheless, the discussion provided in Section 7 suggests that the disparity due to environment geometry can be accounted for with a linear correction. Therefore, we argue that the value of 4 m derived from the *completeness* condition is adequate also to satisfy the atomicity condition.

4 Improving accuracy of estimation for the observed minimum distance r_0

The trajectories provided at the ATR pedestrian group data set[8] are derived by the algorithm of Brščić et al.[9], whose output rate depends on the rate of sensor readings, which may be non-uniform. In order to make the rate of trajectory samples uniform and also to eliminate the effect of gait and sensor noise, we re-sampled the trajectories at 2 Hz. However, the new time resolution can be too sparse for the purpose of computing the observed minimum distance. As a remedy to this issue, we propose interpolating

the position of an individual i between two consecutive time steps t_k and t_{k+1} with its own velocity vector at time t_k, $\mathbf{v}'_i(t_k)$ (see Figure 3).

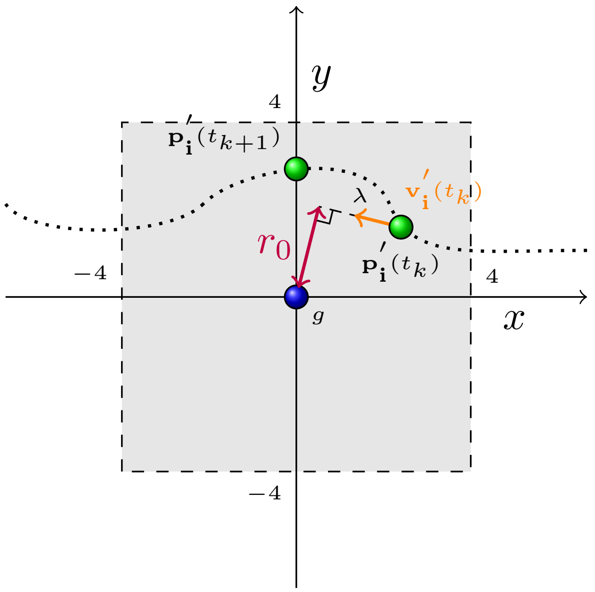

Figure 3. Illustration of computation of the observed minimum distance r_0.

For each time step t_k, the smallest distance between g and i can be computed by measuring the distance from the origin to the line passing through $p'_i(t_k)$, the position of i at t_k and directed by $\mathbf{v}'_i(t_k)$, the velocity of i at t_k. We can consider this distance as the actual minimal distance to the group g in the interval $[t_k, t_{k+1}]$, only if i can reach the position where this distance is observed within $t_{k+1} - t_k$ seconds (0.5 s in this study). We denote the signed distance from $p'_i(t_k)$ to that position with λ, which can be computed by taking the projection of the vector from $p'_i(t_k)$ to the origin (i.e. $-\mathbf{v}'_i(t_k)$) onto the unit vector $\frac{\mathbf{v}'_i(t_k)}{||\mathbf{v}'_i(t_k)||}$,

In explicit terms,

$$\lambda = \frac{-\mathbf{v}'_i(t_k) \cdot \mathbf{p}'_i(t_k)}{||\mathbf{v}'_i(t_k)||}, \tag{3}$$

The distance λ is traveled by i in t_{min},

$$t_{min} = \frac{\lambda}{||\mathbf{v}'_i(t_k)||}.$$

Note that if $t_{min} < t_{k+1} - t_k$, a smaller distance is achieved within the time interval $[t_k, t_{k+1}]$ than at its initial and final instants (t_k and t_{k+1}). Thus, this value is registered as the minimum distance concerning this time interval. This implies that the minimum distance concerning that time interval is achieved at an intermediate instant. Otherwise the lower one of $||\mathbf{p}'_i(t_k)||$ and $||\mathbf{p}'_i(t_{k+1})||$ is registered. In this case, if $||\mathbf{p}'_i(t_k)|| < ||\mathbf{p}'_i(t_{k+1})||$, it means that g and i are getting further away. If $||\mathbf{p}'_i(t_k)|| > ||\mathbf{p}'_i(t_{k+1})||$, it means that g and i are getting closer and that it is highly likely that in the next time interval, they will be even closer. The same operation is carried out for all time intervals and the minimum of all the registered values is used as r_0.

5 Scaling by interpersonal distance

Yücel et al.[19] showed that interpersonal distance between members of a dyad strongly depends on their social relation. For instance, couples were shown to walk with an interpersonal distance significantly smaller than for other social relations (values of interpersonal distance for various social relations and intensities of interaction are show in Table 2).

This variability may affect the behavior of the individual approaching the group. Namely, an individual i may choose not to intrude a group g due to insufficient space (between its members) or may prefer to intrude g due to ample space. So the presence or lack of intrusion may simply be due to geometric circumstances and not stemming from social factors (e.g. strength of social bonding). This effect is actually better studied by measuring distances using a common unit (i.e. meters).

On the other hand, when i does not intrude on g, the effect of the social bonding may be better expressed, if distances are measured with respect to the physical size of the group (interpersonal distance between the members), since avoiding a small group at a distance of, e.g. 2 meters, may involve a stronger avoidance behavior, if the group size is smaller.

Table 2. Average interpersonal distance of groups annotated with each (a) social relation (in ATC data set) and (b) intensity of interaction (in DIAMOR data set).

(a)

Social relation	Average interpersonal distance (mm)
Couples	780
Colleagues	891
Family	935
Friends	835

(b)

Intensity of interaction	Average interpersonal distance (mm)
0 (no interaction)	1115
1	951
2	842
3 (strong interaction)	805

6 Probability of intrusion

In the main track, to study intrusion, we examined the probability of $\bar{r}_0 < 1$, i.e. that the individual reaches a distance from the group center smaller than the average group interpersonal distance. One could argue that an alternative, if not better, definition of intrusion, can be based on studying the probability of having $\bar{r}_0 < 0.5$, i.e. a distance smaller than *the average distance of a group member from the group's center*. For this reason, in this section we perform a similar analysis to the one presented in the main track, but using the value of 0.5. Namely, Figure 4 shows the probability $P(\bar{r}_0 < 0.5)$ and Figure 5 shows the corresponding p-values.

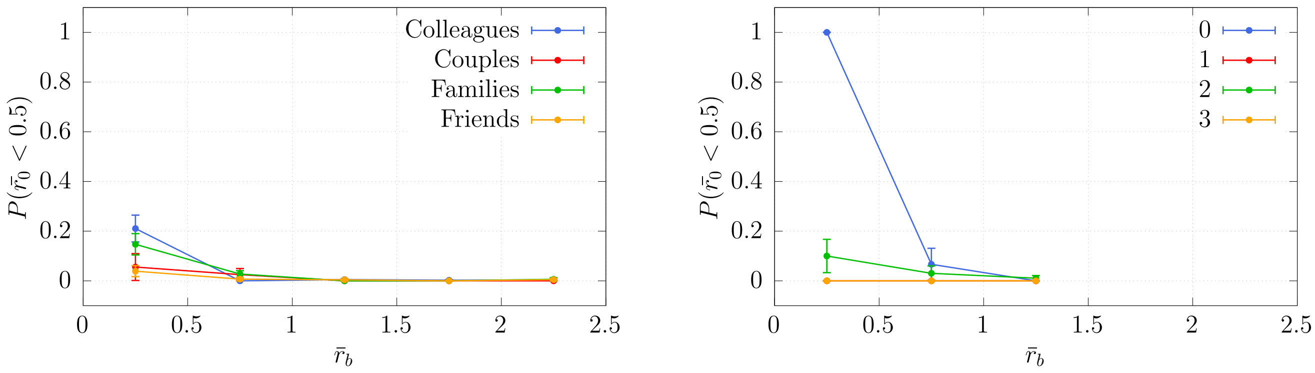

(a) **(b)**

Figure 4. Probability that the distance $\bar{r}_0$ is smaller than 0.5 for (a) for various social relations and (b) intensities of interaction of the group.

Nicely, the probabilities show a similar trend to those given in the main track and confirm our inference that loosely-bonded groups are more likely to be intruded on than strongly-bonded ones.

7 Linear correction to r_b

As mentioned in Section 3, we tried to calibrate the window of observation in such a way that the trajectory of the individual should be close to a straight line for large values of r_b. If the straight-line distance r_b is large, it means that the individual should have enough space to pass comfortably without deviating and we would expect the minimum distance r_0 to be somewhat similar to r_b.

Nevertheless, it seems clear that the curved and narrow nature of the ATC environment puts a limit on the applicability of the straight line hypothesis. In particular, as the width of the ATC corridor is comparable to the size of the chosen window of observation (see Figure 1), we can expect that the environment will pose some constraint on the motion of the pedestrians in particular for large values of r_b.

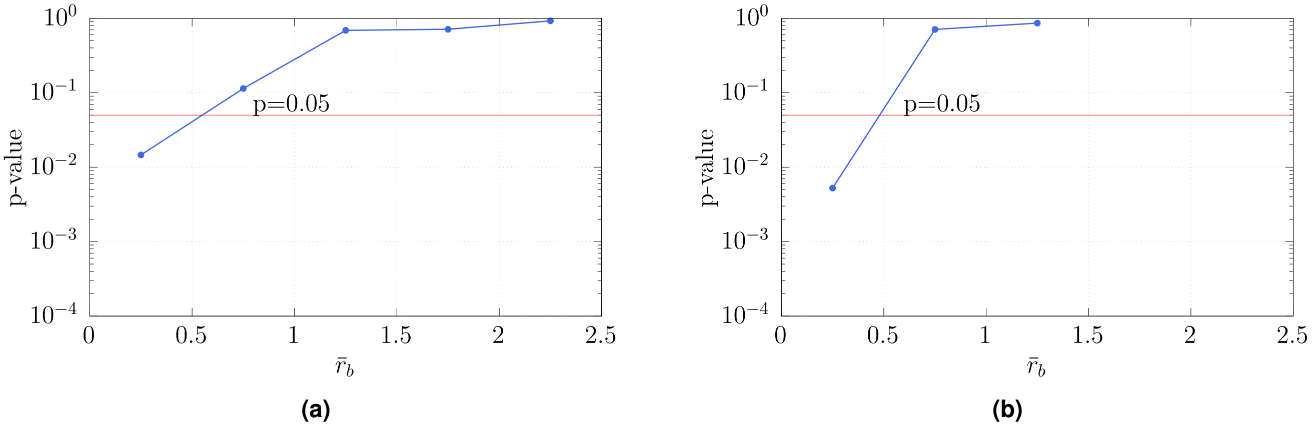

Figure 5. Pearson's χ^2 p-values for the hypothesis of independence of the frequencies of samples verifying $\bar{r}_0 < 0.5$ and $\bar{r}_0 \geq 0.5$ for (a) for various social relations and (b) intensities of interaction of the group.

This is indeed confirmed by the data. Namely, by looking at Figure 6-(a) relating to ATC dataset, we observe that the curves are all noticeably offset from the $x = y$ (dashed) line for high values of $\bar{r}_b$. As $\bar{r}_0$ is smaller than $\bar{r}_b$, it seems as if the individual *steers towards* the group.

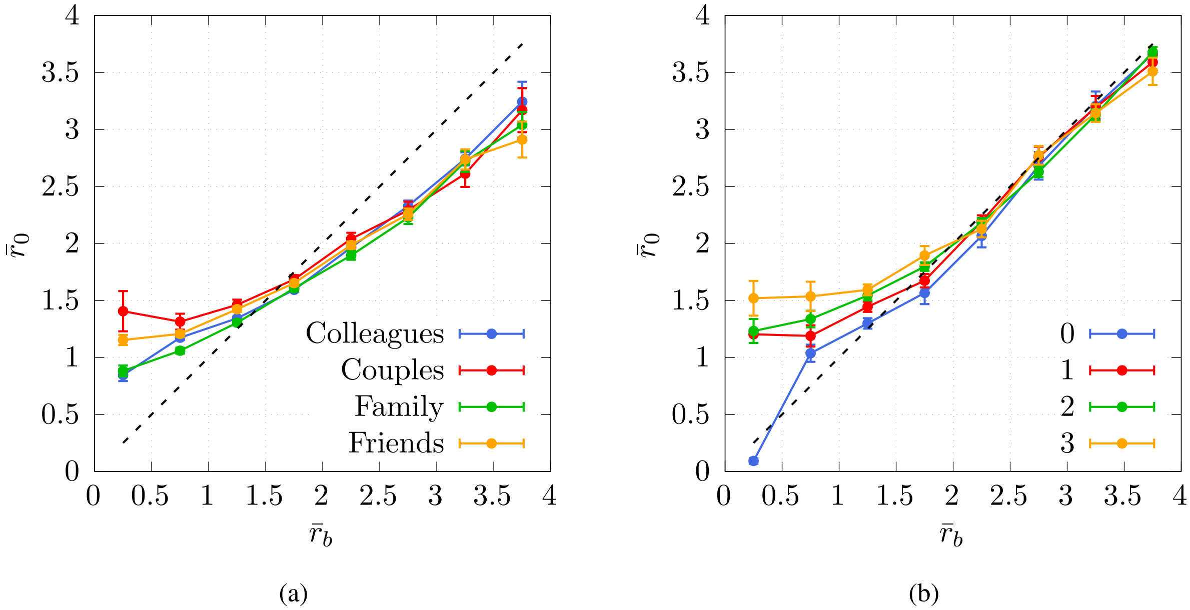

Figure 6. Observed minimum distance $\bar{r}_0$ as a function of the undisturbed straight-line distance $\bar{r}_b$ (a) for various social relations and (b) intensities of interaction of the group. The dashed line corresponds to the $\bar{r}_0 = \bar{r}_b$ linear dependence.

It is not trivial to propose a geometric model for such deviation, due to the relatively complex nature of the ATC environment, but for simplicity's sake we may thus assume the correction on r_b to be linear. Following this hypothesis, we evaluate the impact of environment geometry by computing an average value of the observed distance r_0 (resp. $\bar{r}_0$) for large values of r_b (resp. $\bar{r}_b$) (corresponding to the highest bin in Figure 6), for all groups and individuals. We then compute a correction coefficient c defined as the ratio between the observed average value and the expected value $r_0 = r_b$ (as stated above, when r_b is large, we expect no deviation during encounters). Such coefficients are found to be 0.82 for scaled values and 0.77 for unscaled values. These coefficients can then be used to multiply the values and alleviate the effect of the curvature of the environment. Specifically, when computing the potential U', we replace the values of r_b (resp. $\bar{r}_b$) by the corrected values $r'_b = cr_b$ (resp. $\bar{r}'_b = c\bar{r}_b$).

In Figure 7, we show the scaled distances for groups with various social relations, along with the line corresponding to

the correction coefficient. By definition, the correction fits the various curves for larger values of r_b. For further reference, Figure 8 shows the correction coefficient for the unscaled values concerning all groups and individual pedestrians used for the potential of Figure 7 in the main track. The qualitative agreement between the scaled ATC plots and the unscaled DIAMOR ones suggests that the linear correction used to obtain r_b' and $\bar{r}_b'$ is reasonable.

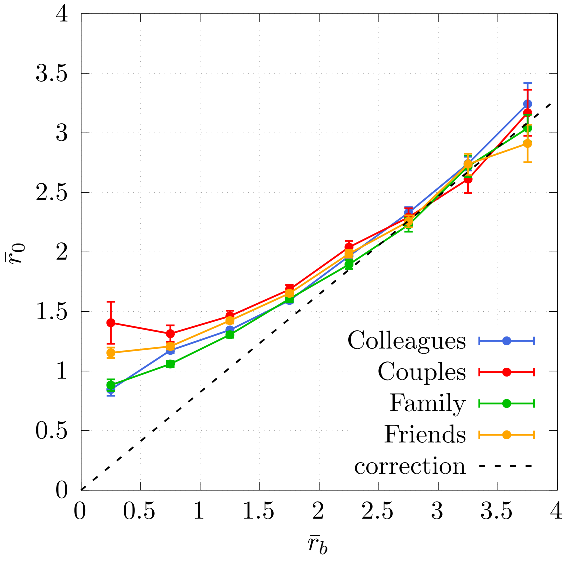

Figure 7. Observed minimum distance against straight-line distance for various social relations (scaled). The dashed black line corresponds to the linear correction applied.

8 Justification of using ANOVA in assessment of statistical significance

To validate the statistical significance of the differences observed in the relation between $\bar{r}_0$ and $\bar{r}_b$ with regard to the social bonding of the group, we performed analysis of variance tests. The standard one-way ANOVA requires the data to confirm three conditions, namely (i) independence of observations, (ii) normality of residuals and (iii) equality of variances. In what follows, we verify and discuss these assumptions.

For (i) independence of observations, we argue that the independence is naturally verified, since the distances are computed for different pairs of groups and individuals, with each group being classified with only one social bonding characteristic.

Regarding (ii) normality of residuals, we carried out D'Agostino's K^2 test on the residuals in each bin (i.e. the distance from which the average values for that bin was subtracted). We found that in 31% of the comparisons (i.e. 5 out of the 16 bins where the test is performed), the assumption of normality was verified (p-value > 0.05).

As for (iii) equality of variances, we used Levene's test to verify that the various samples have equal variance in each bin. We found that the requirement is satisfied in 75% of the cases (p-value for Levene's test > 0.05).

From the above, we inferred that normality of the residuals is the requirement which is not upheld most often, shedding a doubt on the validity of our conclusions. In that respect, we employed an additional statistical test, namely Kruskal-Wallis H-test, which is a non-parametric alternative for the one-way ANOVA. The reason for choosing this test is that it does not require normality of residuals unlike the standard one-way ANOVA. The comparison of the results of Kruskal-Wallis H-test to those of ANOVA is presented in Figure 9. From this figure, one can easily notice that the judgment of statistical significance is not affected by the choice of the test (significance is verified for identical bins). Note that the only bin for which there is a strong difference is $\bar{r}_b \approx 2$ (see Figure 9-(b)), which is nevertheless (in both Figure 9-(a) and (b)) the transition between significant and insignificant.

9 Goodness of fit of the model

Concerning both the scaled and not-scaled distances, the goodness of fit of the proposed models was assessed based on the Akaike information criterion (AIC) and the Kolmogorov–Smirnov (KS) goodness of fit test (see Table 3).

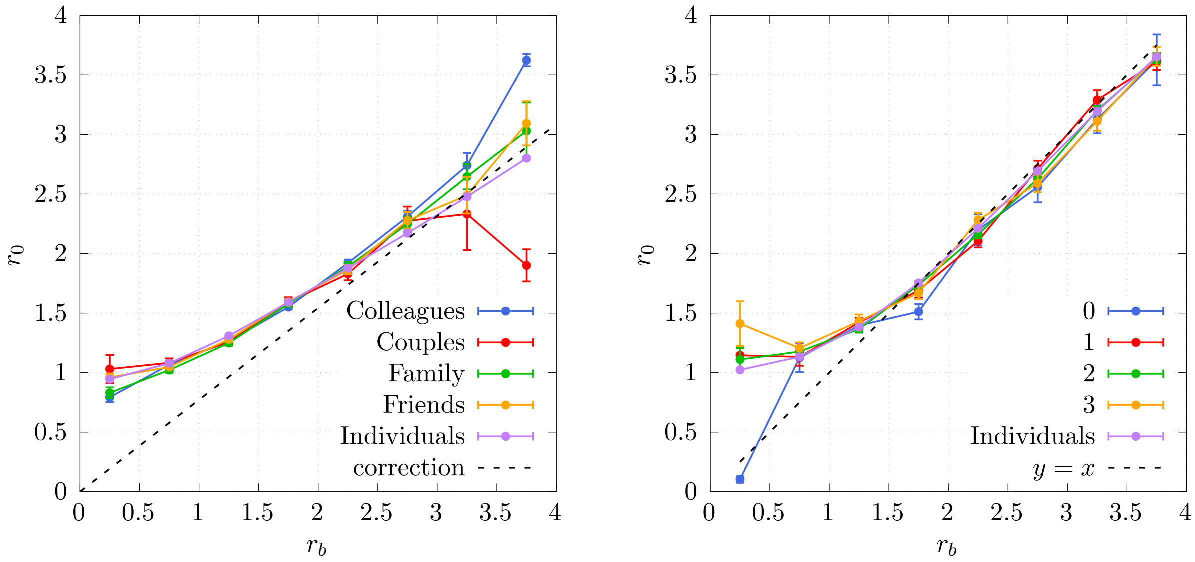

Figure 8. Observed minimum distance r_0 against straight-line distance r_b for (a) various social relations (unscaled) and (b) groups and intensities of interaction (unscaled). The dashed black line corresponds to (a) the linear correction applied for the ATC dataset and (b) to the line $y = x$ for the DIAMOR dataset.

It is hard to interpret the outcomes of AIC concerning a single model, since it is not bounded (it will tend to $-\infty$ when the residual sum of squares gets smaller). Nonetheless, AIC can be used to compare several models, and thus we use it as such. The fit of the model is seen to be relatively poor for non-interacting dyads (see Table 3-(b)) compared to other intensities of interaction and social relations. We also note that the fit is generally better, for scaled distances as compared to the unscaled distances (5 models out of 8).

Concerning the Kolmogorov–Smirnov goodness of fit test, the null hypothesis is that the underlying distributions of the two samples are identical. Our results show that we cannot reject that hypothesis in most cases (note the high p values in Table 3).

For the intensity of interaction of 0 with a p value of 0.02, it is not surprising that the fit is not good, since there is virtually no effect of groups social interaction at that level, as it can be seen in Figure 5-(b) of the main track.

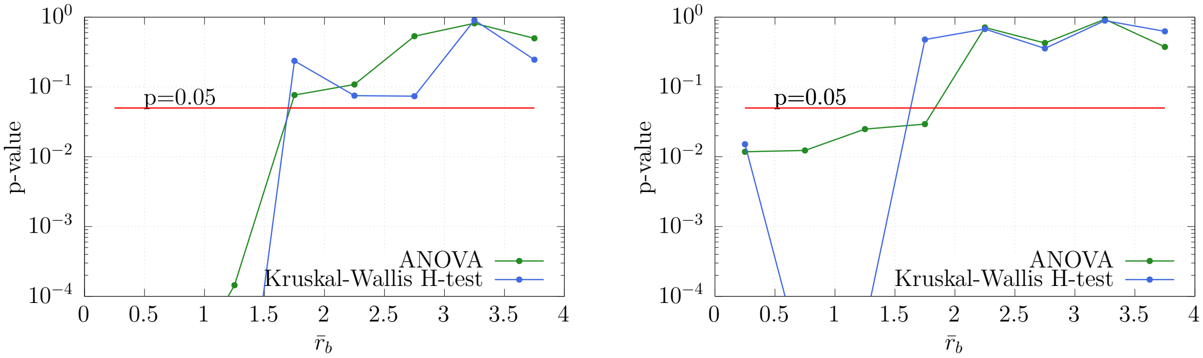

Figure 9. p-values for the ANOVA and Kruskal-Wallis H test of $\bar{r}_0$ (a) for various social relations and (b) intensities of interaction of the dyad. Very low values are not displayed.

Table 3. AIC and Kolmogorov-Smirnov p-values for the goodness of fit of models describing the collision avoidance potential as a function of the distance r of the form k/r^{β} for (a) for various social relations and (b) intensities of interaction of the group.

(a)

Social relation	AIC		KS p-value	
	Not-Scaled	Scaled	Not-Scaled	Scaled
Couples	-8.51	-29.10	0.66	1.00
Colleagues	-27.10	-33.98	0.28	0.98
Family	-50.76	-40.92	0.98	0.98
Friends	-42.72	-39.34	0.98	0.98
Individuals	-44.73	—	0.98	—

(b)

Intensity of interaction	AIC		KS p-value	
	Not-Scaled	Scaled	Not-Scaled	Scaled
0	54.37	57.90	0.02	0.02
1	-27.78	-28.82	0.28	0.28
2	-42.55	-37.55	0.09	0.28
3	-18.22	-29.45	0.28	0.66
Individuals	-48.41	—	0.28	—

References

1. Zanlungo, F., Brščić, D. & Kanda, T. Spatial-size scaling of pedestrian groups under growing density conditions. *Phys. Rev. E* **91**, DOI: 10.1103/PhysRevE.91.062810 (2015).
2. Lui, A. K.-F., Chan, Y.-H. & Leung, M.-F. Modelling of destinations for data-driven pedestrian trajectory prediction in public buildings. In *IEEE International Conference on Big Data*, 1709–1717, DOI: 10.1109/BigData52589.2021.9671813 (Institute of Electrical and Electronics Engineers (IEEE), 2021).
3. Kidokoro, H., Kanda, T., Brščić, D. & Shiomi, M. Simulation-based behavior planning to prevent congestion of pedestrians around a robot. *IEEE Transactions on Robotics* **31**, 1419–1431, DOI: 10.1109/TRO.2015.2492862 (2015).
4. Fahad, M., Chen, Z. & Guo, Y. Learning how pedestrians navigate: A deep inverse reinforcement learning approach. In *IEEE/RSJ International Conference on Intelligent Robots and Systems*, 819–826, DOI: 10.1109/IROS.2018.8593438 (Institute of Electrical and Electronics Engineers (IEEE), 2018).
5. Ono, T. & Kanamaru, T. Prediction of pedestrian trajectory based on long short-term memory of data. In *International Conference on Control, Automation and Systems*, 1676–1679, DOI: 10.23919/ICCAS52745.2021.9649937 (Institute of Electrical and Electronics Engineers (IEEE), 2021).
6. Akabane, R. & Kato, Y. Pedestrian trajectory prediction using pre-trained machine learning model for human-following mobile robot. In *IEEE International Conference on Big Data*, 3453–3458, DOI: 10.1109/BigData50022.2020.9378477 (Institute of Electrical and Electronics Engineers (IEEE), 2020).
7. Kiss, S. H., Katuwandeniya, K., Alempijevic, A. & Vidal-Calleja, T. Constrained gaussian processes with integrated kernels for long-horizon prediction of dense pedestrian crowd flows. *IEEE Robotics Autom. Lett.* **7**, 7343–7350, DOI: 10.1109/LRA.2022.3177849 (2022).
8. ATR. Pedestrian group dataset (2015).
9. Brščić, D., Kanda, T., Ikeda, T. & Miyashita, T. Person tracking in large public spaces using 3-D range sensors. *IEEE Transactions on Human-Machine Syst.* **43**, 522–534, DOI: 10.1109/THMS.2013.2283945 (2013).
10. Zanlungo, F., Yücel, Z., Brščić, D., Kanda, T. & Hagita, N. Intrinsic group behaviour: Dependence of pedestrian dyad dynamics on principal social and personal features. *PLOS ONE* **12**, e0187253, DOI: 10.1371/journal.pone.0187253 (2017).
11. Bugental, D. B. Acquisition of the algorithms of social life: A domain-based approach. *Psychol. Bull.* **126**, 187–219, DOI: 10.1037/0033-2909.126.2.187 (2000).

12. Zanlungo, F., Ikeda, T. & Kanda, T. Potential for the dynamics of pedestrians in a socially interacting group. *Phys. Rev. E* **89**, 012811, DOI: 10.1103/PhysRevE.89.012811 (2014).

13. Bršćić, D., Zanlungo, F. & Kanda, T. Modelling of pedestrian groups and application to group recognition. In *2017 40th International Convention on Information and Communication Technology, Electronics and Microelectronics (MIPRO)*, 564–569, DOI: 10.23919/MIPRO.2017.7973489 (Institute of Electrical and Electronics Engineers (IEEE), 2017).

14. Glas, D. F., Ferreri, F., Miyashita, T., Ishiguro, H. & Hagita, N. Automatic calibration of laser range finder positions for pedestrian tracking based on social group detections. *Adv. Robotics* **28**, 573–588, DOI: 10.1080/01691864.2013.879272 (2014).

15. Glas, D. F., Miyashita, T., Ishiguro, H. & Hagita, N. Laser-based tracking of human position and orientation using parametric shape modeling. *Adv. robotics* **23**, 405–428, DOI: 10.1163/156855309X408754 (2009).

16. Knapp, M. L., Hall, J. A. & Horgan, T. G. *Nonverbal Communication in Human Interaction* (Cengage Learning, 2013).

17. Cinelli, M. E. & Patla, A. E. Locomotor avoidance behaviours during a visually guided task involving an approaching object. *Gait & Posture* **28**, 596–601, DOI: 10.1016/j.gaitpost.2008.04.006 (2008).

18. Kitazawa, K. & Fujiyama, T. Pedestrian vision and collision avoidance behavior: Investigation of the information process space of pedestrians using an eye tracker. In *Pedestrian and Evacuation Dynamics*, 95–108, DOI: 10.1007/978-3-642-04504-2_7 (Springer Berlin Heidelberg, 2009).

19. Yücel, Z., Zanlungo, F., Feliciani, C., Gregorj, A. & Kanda, T. Identification of social relation within pedestrian dyads. *PLOS ONE* **14**, e0223656, DOI: 10.1371/journal.pone.0223656 (2019).
